# Who regulates when told? A systematic investigation of implicit to explicit emotion regulation

**DOI:** 10.3389/fpsyg.2026.1852337

**Published:** 2026-09-15

**Authors:** Julia R. Richmond, K. Maria Nylocks, Karin G. Coifman

**Affiliations:** 1 School of Psychology, Xavier University, Cincinnati, OH, United States; 2 School of Medicine, Emory University, Atlanta, GA, United States; 3 Department of Psychological Sciences, Kent State University, Kent, OH, United States

**Keywords:** emotion, emotion regulation, facial expression, negative affectivity, neuroticism

## Abstract

**Introduction:**

Emotion regulation is a significant area of psychological research that requires investigation of processes that are often difficult for participants to describe, involving implicit and automatic actions. However, research examining emotion regulatory processes often requires individuals to modify their emotional responses in the moment and to report on that strategy use. We aimed to determine if individuals are able to change the regulation of their emotions when implicit goals are made explicit.

**Method:**

Undergraduates (*n* = 101) watched four fear-eliciting films. Between the second and third films, participants were provided psychoeducation on emotion regulation and then, after each of the final two films, participants were asked to report on the emotion regulation strategies they used during those films. Differences in self-reported affect, facial expression, and skin conductance were examined between, before, and after the emotion regulation instruction, as well as between the final two films.

**Results:**

Results suggested little difference in emotion response output following emotion regulation education and strategy reports and that individuals higher in trait negative affectivity were least responsive.

**Discussion:**

Hence, individuals may struggle to respond when implicit regulatory goals become explicit. This has implications for future research, but also for interventions that commonly rely on explicit emotion regulation goals and processes.

## Introduction

Emotion regulation refers to the process by which individuals upregulate or downregulate their emotional experience and responses in the service of goals either deliberately or automatically (Campos et al., 2004; Cole et al., 1994; Gross, 2015; Koole and Rothermund, 2011; Mauss et al., 2007). Dominant models, including the Process Model of Emotion Regulation (Gross, 1998, 2015), typically identify multiple points during emotional contexts in which one can engage in emotion regulation, from prior to the context (e.g., behavioral avoidance of an evocative situation) to shifts in cognition (e.g., reappraisal, acceptance) and attention (e.g., distraction) during and after evocative contexts to modulate emotional experience. Moreover, research over decades has demonstrated that hedonic goals (i.e., reducing discomfort), are most dominant across most individuals and circumstances (English et al., 2017; Larsen, 2000; Riediger et al., 2009). Deficits in negative emotion regulatory processes are broadly implicated in the development and maintenance of most psychiatric disorders (Krueger and Eaton, 2015; Sloan et al., 2017) and thus are increasingly and explicitly targeted in interventions (i.e., Dialectical Behavior Therapy: Linehan, 1993; Emotion Regulation Therapy; Fresco et al., 2013). Hence, research investigating emotion regulatory processes is widespread, with searches of the term “emotion regulation” on Pub Med spawning thousands of articles. Importantly, efforts to define, understand, and modify emotion regulation have largely focused on self-reported difficulties of emotion regulation, self-reported use of emotion regulation strategies during emotional situations, and investigation of instructed vs. spontaneous emotion regulation efficacy.

In examining explicit and implicit emotion regulation processes, it is important to consider what we mean by explicit and implicit. In Braunstein et al., 2017, the authors identify an important distinction between emotion regulation goals and emotion regulation change processes. Regulatory goals can be explicit, when the individual’s primary goal is to regulate their emotions and they are aware of this goal, or implicit, referring to times when an individual does not have a conscious desire or goal to regulate their emotions but instead responds based on general hedonic tendencies (Eldesouky and English, 2018). Further, the emotional change processes utilized can be explicit (e.g., cognitive reappraisal), which requires top-down processing and conscious awareness, or implicit, behavioral regulatory processes that require little top-down control such as attentional shifts or behavioral avoidance that result in improvements in experience (e.g., reduction in discomfort caused by distressing images with visual shifts; Gross and Thompson, 2007). Indeed, evidence suggests that much of the time, emotion regulatory processes occur outside of awareness and have been honed over decades of emotion-linked learning, including operant, associative and symbolic learning processes (Nolen-Hoeksema and Watkins, 2011; Bargh and Williams, 2007).

The distinction between explicit or conscious regulatory efforts versus implicit or automatic regulatory efforts is an important one. Explicit regulatory action often includes volitional and deliberative effort. Indeed, many emotion regulation studies rely on individuals’ ability to report their own spontaneous emotion regulation strategy use or to apply an instructed emotion regulation strategy. In particular, instructed emotion regulation research introduces explicit regulatory goals and relies on explicit regulatory processes (e.g., distraction, suppression, reappraisal). Some findings from these studies suggest that individuals are able to effectively regulate when prompted to do so. For example, Ehring and colleagues demonstrated that depressed patients and healthy controls did not differ in self-report decreased negative affect following engagement in cognitive reappraisal when instructed to use a specific strategy after viewing negatively valenced films (Ehring et al., 2010). Another study demonstrated that dysphoric adults were able to effectively regulate self-reported sadness when instructed to reappraise during a sadness film (Quigley and Dobson, 2014). In a healthy community sample the same results were found, indicating that participants were able to successfully reduce negative emotions through either instructed acceptance or instructed reappraisal during exposure to negative film stimuli (Wolgast et al., 2011). However, other studies have demonstrated mixed results regarding the concordance between self-reported emotion regulation and more objective indices of emotion regulation response (e.g., physiological change: Bernat et al., 2011; Egloff et al., 2006; Grecucci et al., 2020). Because the process of emotion regulation occurs internally, methods to detect emotion regulation processes heavily rely on self-report of processes, however, when these reports do not align with other more objective methods of assessing emotional responding, it is unclear what exactly is occurring.

One important factor to consider in the investigation of emotion regulation is the extent to which emotion regulation is automatic vs. deliberate. Many models of emotion regulation identify both deliberate and automatic processes at play (Mauss et al., 2007; Koole and Rothermund, 2011). Braustein and colleagues identified aspects of emotion regulation that are either automatic or deliberate and serving implicit goals or explicit goals (Braunstein et al., 2017). Indeed, developmental models of emotion argue strongly that conceptualization of one’s emotional experiences, appraisal of events, and even shifts in attention and cognition evolve and develop from infancy through childhood (Beebe and Lachmann, 1998; Cole et al., 1994; Grossmann and Johnson, 2010; Russell, 1980). These early learned tendencies likely become automatic, falling outside of awareness over time, rendering the reliability and utility of self-report of these processes unclear. Because emotion regulation can rely on both automatic and cognitive (deliberate) processes that influence one another, it may be that simply directing individuals to regulate their emotions or instructing them to use a specific emotion regulation strategy is not sufficient to shift variable channels of the emotional response. This could be because responsivity to instruction generally varies between individuals (Jonassen and Grabowski, 1993), the intensity of emotional responses to be regulated can also vary (e.g., negative affectivity: Shackman et al., 2016), and available cognitive resources can vary (e.g., working memory: Coifman et al., 2019). Little research has investigated the efficacy of both implicit and explicit demands to engage in emotion regulation across multiple indices of emotional responding (i.e., subjective, physiological, behavioral). Using these methods is important to determine how and if more explicit emotion regulatory action can influence broader responses of emotion. Further, there is relatively little research on the impact of specific individual differences in responding to implicit -to- explicit demands to regulate, which may better inform the link between deliberate and automatic aspects of emotion regulation.

In order to better understand the efficacy of emotion regulation in response to explicit demands, it may be important to consider factors that may make it more difficult to effectively regulate emotions at all. For example, prior research has demonstrated a link between emotion regulation and response inhibition or cognitive control (King and dela Rosa, 2019; Ochsner and Gross, 2005; Pruessner et al., 2020). Specifically, because individuals need to be able to shift attention and engage in deliberate regulatory processes, individual differences in inhibitory control may determine whether an individual is able to effectively utilize and benefit from instructed emotion regulation strategies, particularly during times of strong emotion. Further, personality traits such as neuroticism (or dispositional negative affectivity) may also impact the efficacy of emotion regulation instruction. Many empirical investigations have found that individuals high in neuroticism may struggle more with use of specific emotion regulation strategies (i.e., reappraisal; John and Gross, 2004; Yang et al., 2020), may be more likely to use strategies considered to be maladaptive (i.e., rumination, suppression repetitive negative thinking; Haga et al., 2009; Mansueto et al., 2022), and may be less able to successfully regulate negative emotions (Harenski et al., 2009).

These individual differences may be important for both laboratory investigations of emotion regulation and psychotherapy, as individuals are often taught many strategies and are tasked to both select and implement those strategies in difficult and emotionally intense situations. This process requires engaging in self-regulation tasks such as (a) identifying the need for regulation, (b) deciding whether and how to regulate, and (c) enacting a strategy (Webb et al., 2012). This may be particularly difficult for individuals seeking treatment, as they are likely to present with high trait negative affectivity and low cognitive control. Thus, the present study may address factors that have important implications for the continued development and refinement of clinical intervention.

## The current investigation

Building on previous work examining the efficacy of implicit vs. explicit emotion regulation, the present study was designed to investigate how emotion regulation information and directed attention to regulation assessed *in vivo* impacts subjective and objective emotional processes and how key individual difference factors such as trait negative affectivity and cognitive control (indexed as response inhibition) may alter this process. The present study examined how implicit goals to regulate emotional responses change when individual attention is directed toward regulation, and then individuals are provided with examples as to how to regulate (via strategy lists), systematically, across a fear-inducing task. Specifically, we utilized a fear-reactivity paradigm where participants watched a series of 4 films. In order to determine the influence of various types of emotion responding, the present study utilized a multimethod approach that incorporated not only self-reported affect, but also physiological responding (i.e., skin conductance) and facial expressions of emotions (see Figure 1 for a schematic of the study design). During the first 2 films, participants were not given any instruction to modulate their emotional experience, but were expected, given the precise sequence of films to do so spontaneously given the presumed dominance of hedonic motivation. Prior to the third film, participants were provided, for the first time, with a brief description of what emotion regulation is but were not provided with specific instructions to regulate their emotions in a particular way. After the third film, participants were provided with a checklist of emotion regulation strategies and were asked to indicate which strategies they utilized. We considered this checklist as an emotion regulation “prime” that may impact strategies the participants reported during the fourth film by introducing more explicit emotion regulatory processes. As all participants engaged in all parts of the task, this investigation sought to systematically evaluate how emotion responses changed within-participants, following simple emotion regulation psychoeducation, across multiple response channels: self-report of experience, coded facial expressions, physiological responding.

**Figure 1:**
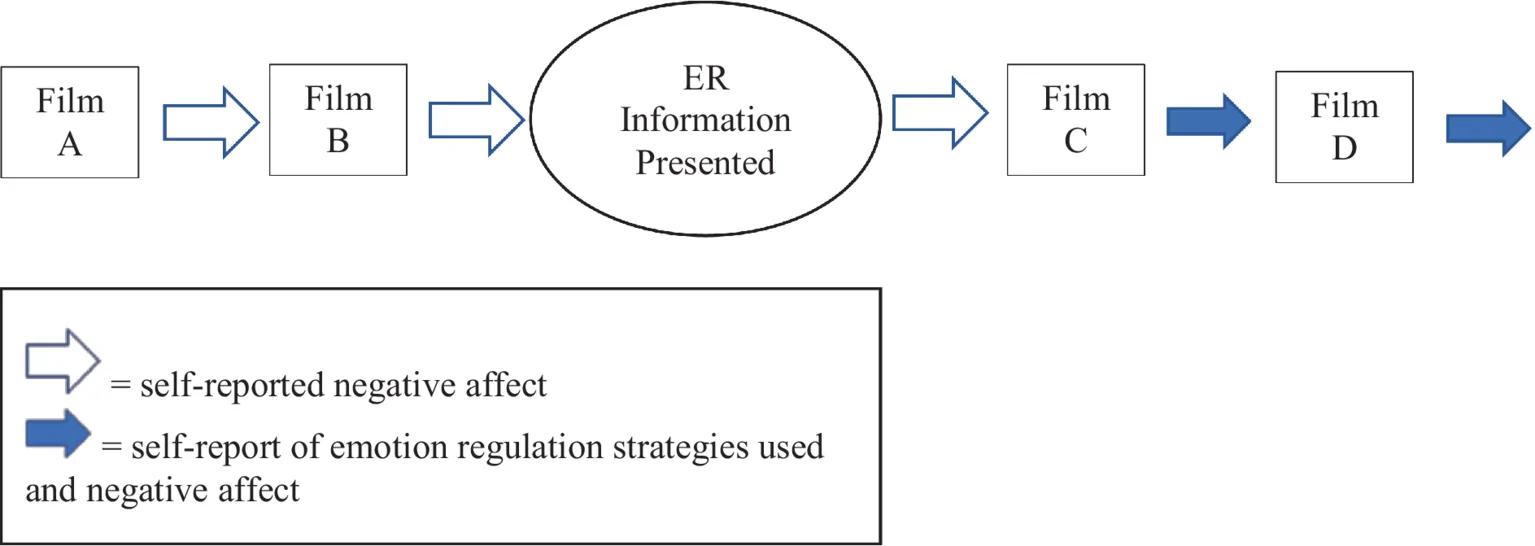
Study design.

Based on prior research examining the efficacy of explicit emotion regulation strategies (e.g., cognitive reappraisal; Mauss et al., 2007; Ochsner and Gross, 2008), we hypothesized that providing participants with an emotion regulation checklist would improve regulatory efficacy and decrease negative emotional responses to fear-inducing films, as the presentation of this checklist was expected to transition participants from implicit regulatory goals to explicit regulatory goals. We expected this would be evident as reductions in self-reported negative affect/fear, decreased negative/fear facial expressions, and reduced physiological reactivity. More specifically, we expected that participant responses to the two films prior to emotion regulation information would be characterized by greater emotional reactivity than responses to the two films following the presentation of emotion regulation information. Further, we expected greater emotional reactivity during the third film (when participants are only directed to think about emotion regulation) when compared to the fourth film (after participants are provided with a list of regulatory strategies), as evidence that the suggested emotion regulation strategies encouraged more effective regulation.

## Methods

### Procedure

Participants were undergraduate students recruited throughout two academic semesters from the Psychology department subject pool at a large public university in the Midwest in the United States. All participants provided written informed consent prior to study. Participants were asked to complete a number of self-report questionnaires (e.g., demographic information, trait affectivity symptoms), a computerized response inhibition task, and then a fear-regulation task containing four emotionally evocative film clips. During the film clips, participants were monitored for skin conductance and were video recorded. Participants were informed during the consent process that they would be video recorded. Once the laboratory session was complete, participants were compensated with research credits. The present study was approved by the university’s institutional review board.

### Transparency and openness

Sample selection, size and power estimation were determined a-priori for a separate project (Nylocks, 2020) with distinct aims. This investigation is a novel analysis of an existing dataset. The original study had determined that a sample of 100 participants would be sufficient. However it may be that the current investigation is underpowered to detect small effects. No data included in this investigation has been published. Data analyses were pre-registered^1^ and all data and code are in the same repository. Materials are included in the supplemental materials.

### Participants

Participants were *N* = 101 undergraduate students (71.3% Caucasian, 76.2% female, *M*age = 20.09 years, SD = 3.50). In order to be eligible for the study, participants needed to be at least 18 years of age and be fluent in the English language. No additional exclusion criteria were established.

### Measures

#### Trait negative affectivity

In order to obtain an index of dispositional negative affectivity, an aggregate was derived from three (standardized) instruments (average correlation of *r* = 0.50, *p* < 0.001), per prior research (Craske et al., 2012).

*The State–Trait Anxiety Inventory* (STAI; Spielberger et al., 1983) The 20 trait-level items only were used in the current study. Scores were summed in order to generate an index of general anxiety. Possible scores ranged from 20 to 80, with scores in the present sample ranging from 23 to 68. The mean score for the current sample (*M* = 43.51, SD = 10.56) indicates moderate levels of general anxiety in the current sample (consistent with other college student samples – Vigneau and Cormier, 2008). Internal consistency for this sample was adequate (α = 0.91).

*The Symptoms Checklist-90-Revised* (SCL-90-R) is a widely used measure of psychological distress (Derogatis and Melisaratos, 1983). The present study only utilized the 10-item Anxiety subscale, and an average score was calculated for each participant. Possible scores on this measure range from 0 to 4, with scores in the present sample ranging from 0 to 3.25. The mean score for this sample was (*M* = 0.77, SD = 0.66) and the *T* score of this sample was 59.33, which indicates clinically significant albeit mild anxiety and slightly greater reported distress in the current sample as compared to the initial non-clinical adult sample (Todd et al., 1997). However, this mean is similar to other undergraduate samples (*M* = 0.72; Barker-Collo, 2003). Internal consistency for this sample was adequate (α = 0.86).

*The Behavioral Inhibition Scale* (BIS; Carver and White, 1994) was used to measure behavioral inhibition. The current study utilized the 7-item BIS subscale of this measure, with possible scores ranging from 7 to 28. In the present sample, the range was 9–28 and the average score on the BIS subscale was similar to scores from other undergraduate samples (*M* = 21.22, SD = 4.18; Wardell et al., 2011). Internal consistency was adequate (α = 0.82).

#### Fear regulation task

Participants were asked to engage in a task involving viewing four, fear-evoking evocative film clips. Each fear-eliciting video was approximately 90 s long, and every video was followed by a one-minute-long recovery period to allow for spontaneous recovery. Following recovery periods participants were shown a positive emotion inducing clip to normalize their responses (see Figure 1). This process of eliciting changing emotional responses by video-induced contextual shifts has been used in multiple prior paradigms evaluating spontaneous emotion processing with validity and reliability (e.g., Coifman et al., 2016, 2019). During this task, participants’ emotional responses were recorded through multiple methods (self-reports of emotional experience after each film and each recovery period; high-resolution recordings of emotion facial expression; and real-time recording of skin conductance). Film clip presentation order was randomized (fear clips were randomized; positive clips were randomized) between participants. Films used during this task depicted: (1) a doctor examines an infected wound on the back of a woman and spiders crawl out of the wound while the woman screams (*Fear Clinic,* Hall, 2014), (2) a woman wakes up to find a demon-like creature next to her bed (*Bedfellows*, youtube.com), (3) in a dark house, limbs extend through the walls and ceiling forming a monster or demon-like creature (*Real Demons*, youtube.com), (4) a large spider suddenly jumps down from the ceiling onto a man standing on a ladder (*Spider Attacks Daddy*, youtube.com). All selected video clips were evaluated as part of an online pilot study, *n* = 271, using methods consistent with prior validation of video stimuli (Gilman et al., 2017). The pilot study demonstrated that these four video clips elicited significant levels of fear, details are in the supplemental materials (see Supplementary Table S1).

Half-way through the task, following the second fear-eliciting clip (“Clip B”), all participants were presented with general information defining and explaining emotion regulation and were prompted to consider how they might be regulating their emotions in the clips that followed (“C” and “D”). The goal of the initial prompt was to bring emotion regulatory processes into awareness. Instructions utilized in the current study were adopted from Gross (1998) and have been used in previous emotion elicitation research using video clips (Gilman et al., 2017). Specifically, participants were told: “As before, when you watch the films, you may have some emotional responses. This time, however, as you watch we also want you to notice in particular the intensity of your emotions or feelings and any things you might be doing to change the intensity of your emotions. For example, sometimes, you may opt to increase your feelings and choose to do certain things, think certain things that enhance your emotional responses. Other times, you may opt to decrease your feelings and choose to do certain things, think certain things that reduce your emotional responses. Changing your emotional response in these ways is called emotion regulation and in this part of the task we will be asking you to attend to any activities that you might or might not choose to do, during and after each film.”

Participants were prompted to describe whatever regulatory action was taken, with an open-ended prompt following the recovery period from the third (“C”) film. To do this they spoke into a microphone for 30–60 s and responses were transcribed and coded (described below). Participants were not presented with examples of emotion regulation strategies (e.g., via an emotion regulation checklist) until after they viewed the third film (“C”) and after their open-ended response. At that point, participants were presented with a checklist of strategies (described below) and asked to indicate on this checklist what strategies they employed during the viewing of “C.” Participants completed this same process, open-ended prompt response and checklist after viewing the fourth video (“D”). All study materials are included in the supplemental materials and Figure 1 is a complete schematic of the study design.

#### State emotion measurement

##### Self-reported negative emotion

Participants were prompted to report negative emotional experience immediately following each film clip and immediately following each recovery period by rating emotion-words on a 7-point Likert scale. Emotion-words were fear, sadness, disgust, guilt, distress, and anger, consistent with dominant affective circumplex models of affect (Rafaeli et al., 2007). Ratings of these negatively valenced words were aggregated to generate a mean negative affect score for each participant for each video clip. Internal consistency for this sample was good across each film clip (αs = 0.98–0.99). Mean self-reported negative affect was calculated for each segment (see Supplementary Table S3). In addition, participant’s self-report of fear, specifically, was also examined separately in the current study, to test for unique impacts of fear emotion, given the specific elicitation of fear in the videos.

##### Skin conductance

EDA (electrodermal activity) was collected in real-time but segmented to capture periods coinciding with before, during, and after each film clip. Average EDA during each time point (baseline, film clips, recovery periods) was used to determine sympathetic autonomic responsivity. The EDA signal was acquired through a 31-channel A/D converter operating at a resolution of 12 bits and with an input range of 2.5 V to +2.5 V. Amplification rates, high-pass filter (HPF), and low-pass filter (LPF) settings were as follows: ECG (gain = 500, HPF = 0.1 Hz, LPF = 1,000 Hz) and EDA (gain = 0.1 V/S, HPF = none/DC, LPF = 10 Hz, 6 dB/octave, single pole RC) (Mindware Technologies, Gahanna, OH). EDA data were cleaned and artifacts removed using customized Bio-Lab™ Software (MindWare Technologies, Gahanna, OH) and visual inspection of artifacts. Problematic segments of the EDA wave were excluded following visual inspection, and *n* = 18 participants were excluded from analyses because of equipment malfunction and administrator error (*n* = 13) and lack of detectable signal due to low natural skin conductance (*n* = 5; see Supplementary Table S2). EDA scores were significantly positively skewed, thus scores were normalized using a log transformation (Braithwaite and Watson, 2015). Then, EDA scores were person-centered to account for natural increases in EDA signal across the lengthy task period (i.e., “drift” Tronstad et al., 2010) and to isolate unique reactivity to each context (Coifman et al., 2019). These person-centered scores reflecting sympathetic nervous reactivity were then used in all analyses. Logarithmic transformed mean skin conductance level across the full fear regulation task for the current sample was M = 0.48, SD = 0.42. Raw mean skin conductance response (SCR) values and transformed, person-centered SCR values for the duration of the baseline period, for each film clip and for each recovery period, are presented (in uSiemens) in Supplementary Table S4. Transformed, person-centered values were used in all analyses.

##### Emotion facial expressions

High-resolution recordings of participant faces during each film clip and recovery from each film clip were used to index specific discrete emotional expressions. Noldus Facereader version 7.1 (Leesburg, Virginia; Loijens et al., 2014), an automated facial coding software derived from the standard Facial Action Coding System (Ekman and Friesen, 1978) was used. This software uses 3D modeling and deep learning algorithms to detect changes in facial muscle contractions associated with the expressions for basic emotions (e.g., fear, anger, sadness, disgust), assigning each emotion a score between 0 and 1 at a rate of 30 frames/s (Mitchell et al., 2022). As in the self-reported emotional response, we included an aggregate index of negative emotional expressions as well as an index of fear only expression. Hence scores for both fear expression and the negative emotional expression aggregate were derived from recordings at each time point (during each film clip, each recovery period). Problematic segments due to failed recording or participant movements (e.g., ducking head out of the frame) were excluded and *n* = 4 participants were not included in these analyses. Supplementary Table S5 presents the means for all segments.

#### Emotion regulation strategy use

Participants completed an emotion regulation strategy checklist following film C and following film D only. The checklist was derived from a validated checklist of emotion regulation strategies (Heiy and Cheavens, 2014). Although this measure was developed for use in experience sampling methods, utilization of similar emotion regulation strategy checklists is common in previous research (Ehring et al., 2010; Szasz et al., 2011; Volokhov and Demaree, 2010). This checklist assessed for use of 16 emotion regulation strategies, including: acceptance, distraction, rumination, denial, emotional suppression, expressive suppression, generalizing, blaming others, blaming self, consequences, problem-solving, cognitive reappraisal, positive refocus, benefit finding, perspective, and sleep. Participants were instructed to check off each statement that was a good representation of what they did in response to the video. Number of strategies endorsed per video ranged from 1 to 10. On average, participants reported use of 3.49 (SD = 1.75) strategies from the checklist during Film C and 3.41 (SD = 1.90) strategies during Film D. Number of strategies did not significantly differ following presentation of the checklist *t*(92) = 0.53, *p* = 0.60, *d* = 0.06. The sum of strategies endorsed was used in analyses.

Participants also provided open-ended descriptive responses of what they might have done to regulate emotion after viewing film C and D. This was prior to completing the checklist described above. These open-ended responses were recorded using a microphone and then later transcribed by blinded research assistants and coded according to categories that matched the Heiy and Cheavens checklist measure (see Supplementary materials). For example, participants reported engaging in avoidance, cognitive reappraisal, distraction, rumination, denial, expressive suppression, emotional suppression, problem-solving, acceptance, positive refocus, perspective taking, and considering consequences. On average, participants provided open-ended responses indicating they engaged in 1.32 (SD = 0.67) strategies during Film C and 1.29 (SD = 0.73) strategies during Film D. Number of open-ended strategies reported did not differ following presentation of the checklist, *t*(92) = 0.68, *p* = 0.50, *d* = 0.07. The sum of strategies reported was used in analyses.

#### Emotional Stroop task

A traditional Color-word Stroop task (Stroop, 1935) was adapted to measure response-inhibition, or the ability to suppress a dominant or pre-potent response, within an emotional context. Instead of altering the target stimuli (i.e., color words), the background screen was altered to be explicitly emotional. This then forces the participant to maintain attention on the neutral color word stimuli, down regulating the interference from the emotional material behind it. This method has been used in other investigation of cognitive control processes (e.g., emotional working memory: Coifman et al., 2019) and has been proven highly predictive of spontaneous emotion regulatory action both in lab and in daily life, across clinical, college, and community samples (e.g., Coifman and Aurora, 2022; Seah and Coifman, 2022). Here the adapted Stroop task contained three sets of 80 trials each, and each set presented 25% congruent and 75% incongruent trials. In this modified version of the Color-word Stroop task, each word was presented superimposed over an emotionally evocative picture. All pictures included in the task were selected from a valid picture set (International Affective Picture Set; Bradley and Lang, 2007) and remained on the screen for 500 ms. Each of the three-sets contained 20 images that would appear four times each, and five images that would appear five times each. Specifically, one set included 20 negatively valenced images randomized to appear throughout the set, one set included 20 positively valenced images, and one set included 20 neutral images. Lastly, between each set of trials, participants passively viewed images of neutral landscapes presented on the screen for 10 s each.

Data was cleaned by removing data when the reaction time for a trial exceeded the participant’s own mean response time for that condition by 3 SD or more. If the reaction time for a trial was below 200 ms, it was also removed. This resulted in dropping less than 1% from the current sample. Scoring here was consistent to traditional Color-word Stroop task scoring in that for each participant, mean reaction time scores were calculated for incongruent and congruent trials. Then, the congruent mean was subtracted from the incongruent mean in order to generate interference scores for each participant (Strauss et al., 2005). Participants with reaction times greater than 3 SD from the mean were excluded from analyses (*n* = 3). Mean reaction times for congruent and incongruent sets are presented in Supplementary Table S6. Difference scores within the negative picture set were used in the present analyses.

#### Film-task manipulation check

First, a series of ANOVAs were conducted to confirm no effects of the order of films, despite the random presentation. Results suggested that film presentation order did not significantly impact self-reported affect, skin conductance, or facial expression outcomes, *F*s < 2.32, *p*s > 0.07. Paired samples *t*-tests were conducted to determine whether self-reported negative affect, EDA, and facial expressions were significantly affected by each film. Analyses revealed significant differences between self-reported negative affect after each film, in relation to the recovery period that followed (see Figure 2) to confirm the efficacy of each film elicitation and the expected hedonically motivated shifts. Specifically, there were significant differences between film A (*M* = 1.98, *SD* = 0.83) and a subsequent recovery period (*M* = 1.45, *SD* = 0.64), *MD* = 0.53, *t*(90) = 6.51*, p* < 0.001, 95% CI [0.33, 0.61], *d* = 0.68, between film B (*M* = 1.89, *SD* = 0.89) and subsequent recovery period (*M* = 1.45, *SD* = 0.58), *MD* = 0.44, *t*(89) = 5.81, *p* < 0.001, 95% CI [0.29, 0.58], *d* = 0.61, between film C (*M* = 2.16, *SD* = 0.83) and following recovery period (*M* = 1.48, *SD* = 0.57), *MD* = 0.71, *t*(93) = 9.62, *p* < 0.001, 95% CI [0.54, 0.82], *d* = 0.99, and between film D (*M* = 2.08, *SD* = 0.90) and the final recovery period (*M* = 1.50, *SD* = 0.61), *MD* = 0.63, *t*(92) = 7.91, *p* < 0.001, 95% CI [0.43, 0.72], *d* = 0.82.

**Figure 2:**
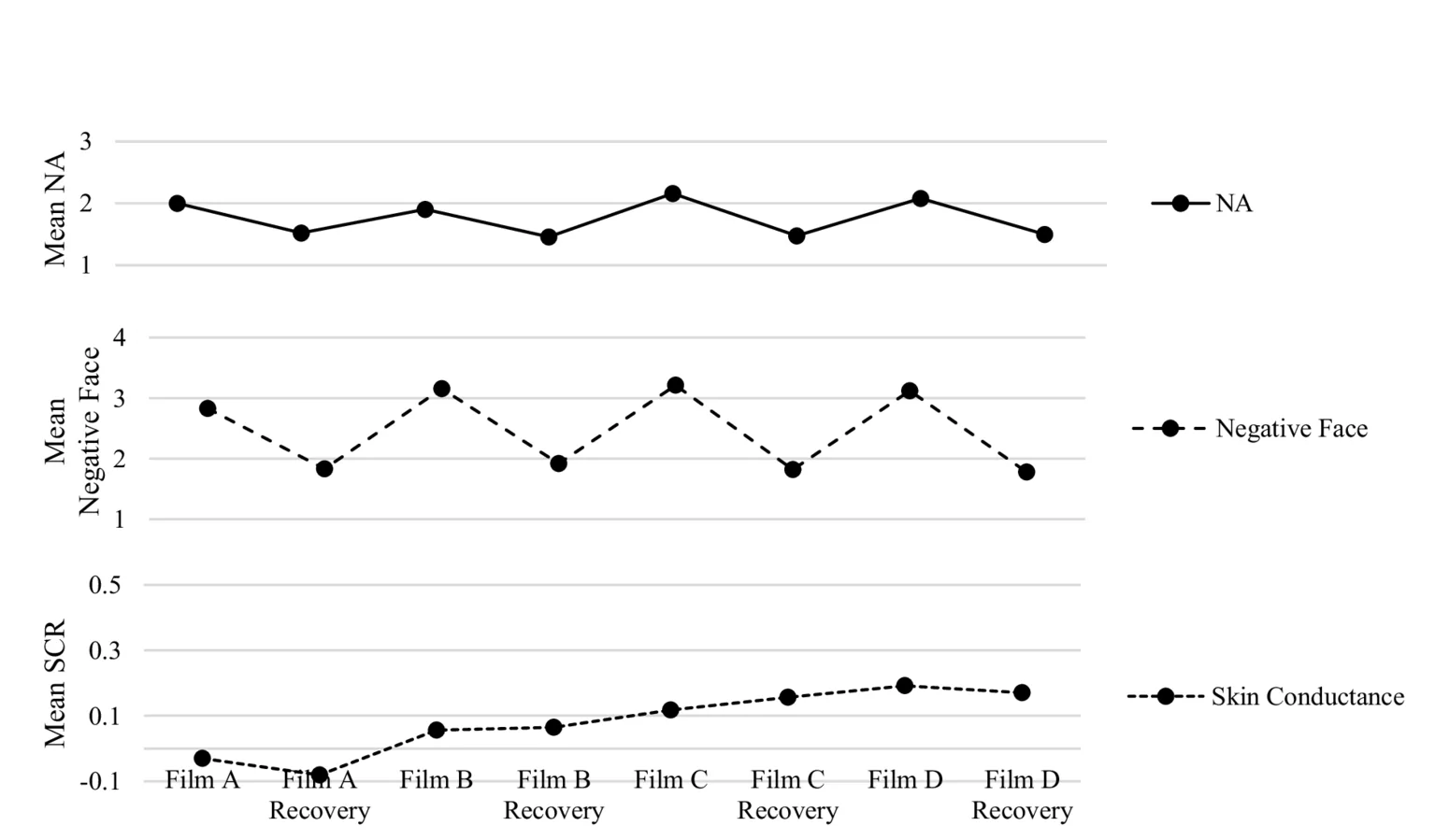
Means of negative affect, negative facial expression, and skin conductance across all films.

These findings were replicated when examining facial expression data. Facial expressions during each film were significantly different from facial expressions during a subsequent recovery period. Negative facial expressions during film A (*M* = 2.83, *SD* = 1.47) were significantly greater than during a recovery period (*M* = 1.83, *SD* = 0.75), *MD* = 0.93, t(88) = 7.20, *p* < 0.001, 95% CI [0.72, 1.27], *d* = 0.76, negative facial expressions during film B (*M* = 3.04, *SD* = 1.49) were significantly greater than during the recovery period (*M* = 1.88, *SD* = 0.75), *MD* = 1.17, t(86) = 9.15, *p* < 0.001, 95% CI [0.96, 1.49], *d* = 0.98, negative facial expressions during film C (*M* = 3.05, *SD* = 1.49) were significantly greater than those during the recovery period (*M* = 1.78, *SD* = 0.64), *MD* = 1.27, *t*(86) = 8.95*, p* < 0.001, 95% CI [1.05, 1.65], *d* = 0.96, and negative facial expressions during film D (*M* = 3.05, *SD* = 1.40) were significantly greater than those during the final recovery period (*M* = 1.78, *SD* = 0.58), *MD* = 1.27, *t*(79) = 8.91, *p* < 0.001, 95% [0.99, 1.56], *d* = 0.99.

Further, these findings were largely replicated when examining skin conductance, as EDA during film A (*M* = −0.09, *SD* = 0.66) and post-film A recovery (*M* = -0.55, *SD* = 0.87) were significantly different, *MD* = 0.47, *t*(80) = 4.51, *p* < 0.001, 95% CI [0.36, 0.67], EDA during film D (*M* = 1.73, *SD* = 1.69) was significantly different from post-film D recovery (*M* = 1.48, *SD* = 1.68), *MD* = 0.25, t(85) = 3.45, *p* < 0.001, 95% CI [0.11, 0.39], *d* = 0.37. However, there was not a significant difference between EDA during film B (*M* = 0.57*, SD* = 0.70) and film B recovery (*M* = 0.61, *SD* = 0.71), *MD* = −0.04, *t*(89) = −0.51, *p* = 0.61, 95% [−0.20, 0.12], *d* = 0.05. Further, there was a significant difference between EDA during film C (*M* = 1.02, *SD* = 0.1.32) and recovery following film C (*M* = 1.32, *SD* = 1.31), but the direction of this difference was opposite to expectation, *MD* = −0.30*, t*(90) = −3.68, *p* < 0.001, 95% CI [−0.46, −0.14], *d* = 0.39.

### Missing data

There were 31 participants with missing data. See Supplementary Table S2 for details regarding missing data. A pairwise deletion approach to missing data was used in order to conserve the sample size. Thus, the degrees of freedom vary slightly across final analyses. Participants with missing data were not found to be different from the main sample on any key study variables, including age, sex, psychological symptoms, Stroop interference scores, fear expression, positive emotion expression, or skin conductance.

### Analysis plan

These analyses were pre-registered^2^. To identify covariates for primary analyses, correlation analyses and independent t-test were conducted to examine relations of dependent variables with relevant demographic characteristics (i.e., age, sex).

The primary analyses were a series of repeated measures ANCOVA conducted to test each emotion response index as an outcome. Each outcome (self-reported affect, physiological reactivity, emotional expression) was tested in two ways. First, models were conducted comparing the first set of films (films A&B; pre-instruction about emotion regulation; see Figure 1) to the second set of films (films C&D; post-instruction) to test the impact of introducing emotion regulation as a concept and directing attention to emotion regulatory processes on spontaneous emotional responses. Then, emotion outcomes (i.e., self-reported affect, skin conductance, and facial expressions) were tested comparing films C & D within the second set, to determine the impact of introducing explicit emotion regulatory strategies to participants.

Due to the number of primary analyses conducted, we used the Benjamini-Hochberg False Discovery Rate (FDR) Correction procedure to correct for Type I error (Benjamini and Hochberg, 1995). *p*-values were placed in ascending order and are then assigned a corrected level of significance, which is calculated using the formula (i/m)*q, where i represents the *p*-value’s rank, m represents the number of hypothesis tests, and q is the initial alpha level (0.05). We conducted this procedure for 2 sets of 5 tests: (1) the 5 ANCOVAs examining differences between films A&B compared to C&D, and (2) the 5 ANCOVAs examining differences between film C compared to film D. The results of this approach indicated that tests in both clusters will be considered significant at *p* < 0.01. Following these planned, pre-registered, analyses, changes in emotional reactivity during recovery periods were also explored to determine the impact of emotion regulation instructions on emotional recovery following the films. Specifically, changes in emotion from film C, a recovery period following film C, film D, and the recovery period following film D were explored. Next, we examined associations of open-ended descriptions of emotion regulation activity (during films C and D), and strategies selected (following films C and D), to emotion output in those same segments. Finally, we ran a series of moderation analyses to explore the potential role of trait negative affectivity and response inhibition (derived from the emotion Stroop performance) in these outcomes over time.

## Results

### Preliminary analyses

Descriptive data on and correlations among the primary study variables are presented in Table 1. Analyses examining associations between demographic characteristics and outcome variables revealed a significant difference in negative facial expressions as a function of participant sex during the first two films, *t*(86) = −2.15, *p* = 0.04, *d* = 2.58, 95% CI [−2.71, −0.10], as well as the second two films, *t*(80) = −2.26, *p* = 0.03, *d* = 2.41, 95% CI [−2.63, −0.17], such that females displayed significantly greater negative emotional expression than males. Further, there was a significant difference in self-reported negative affect during the final film as a function of sex, *t*(91) = −2.10, *p* = 0.04, such that females reported significantly greater negative affect than males. There were no significant correlations between study variables and participant age. Thus, participant sex was included as a covariate in all analyses. These sex differences are consistent with previous research on emotion expression and experiences between the sexes, which demonstrates that females are more likely to display both positive and negative facial expressions (Kring and Gordon, 1998) and are more likely to self-report stronger negative emotions than males, particularly when reporting on fear-spectrum emotions (i.e., anxiety; Campbell et al., 2021; Choi et al., 2026).

**Table 1:** Correlations between study variables.

	Mean (SD; n)	1. SR NA films AB	2. SR NA films CD	3. SR fear films AB	4. SR fear films AB	5. PC SC films AB	6. PC SC films CD	7. Neg face films AB	8. Neg face films CD	9. Fear face films AB	10. Fear face films CD	11. Neg affectivity
1. SR NA films AB	3.96 (1.48; 88)	--	--	--	--	--	--	--	--	--	--	--
2. SR NA films CD	4.24 (1.51; 89)	0.562^***^	--	--	--	--	--	--	--	--	--	--
3. SR fear films AB	3.09 (1.54; 92)	0.836^***^	0.522^***^	--	--	--	--	--	--	--	--	--
4. SR fear films CD	3.38 (1.72; 93)	0.506^***^	0.868^***^	0.583^***^	--	--	--	--	--	--	--	--
5. PC SC films AB	0.47 (0.68; 82)	0.183	0.210	0.262^*^	0.207	--	--	--	--	--	--	--
6. PC SC films CD	2.72 (2.93; 85)	−0.204	0.011	−0.163	−0.018	0.054	--	--	--	--	--	--
7. Neg face films AB	5.99 (2.63; 86)	0.546^***^	0.296^**^	0.541^***^	0.252^*^	0.160	−0.112	--	--	--	--	--
8. Neg face films CD	6.22 (2.48; 80)	0.259^*^	0.389^***^	0.324^**^	0.394^***^	0.092	0.005	0.673^***^	--	--	--	--
9. Fear face films AB	4.24 (2.16; 86)	0.441^***^	0.230^*^	0.500^***^	0.192	0.297^**^	−0.026	0.833^***^	0.592^***^	--	--	--
10. Fear face films CD	4.52 (2.19; 80)	0.248^*^	0.422^***^	0.348^**^	0.444^***^	0.196	0.044	0.556^***^	0.857^***^	0.602^***^	--	--
11. Neg affectivity	0.029 (0.81; 97)	0.045	0.127	0.008	0.093	0.001	−0.164	0.068	0.189	0.081	0.155	--
12. Neg Stroop	56.13 (127.36; 95)	−0.057	0.024	−0.043	−0.001	−0.124	−0.004	0.072	0.060	0.042	0.134	−0.046

SR NA Films AB = self-reported negative affect during films A and B, SR NA Films CD = self-reported negative affect during films C and D, SR Fear Films AB = self-reported fear during films A and B, SR Fear Films CD = self-reported fear during films C and D, PC SC Films AB = person-centered skin conductance during films A and B, PC SC Films CD = person-centered skin conductance during films C and D, Neg Face Films AB = negative facial expressions during films A and B, Neg Face Films CD = negative facial expressions during films C and D, Fear Face Films AB = fear facial expressions during films A and B, Fear Face Films CD = fear facial expressions during films C and D, Neg Affectivity = standardized trait negative affectivity, Neg Stroop = interference score on negative affect Stroop task. *p < 0.05, **p < 0.01, ***p < 0.001.

### Primary analyses

#### Self-reported negative affect

Results of repeated measures ANCOVAs examining the change in self-reported negative affect and fear between films A&B and C&D were not significant, *F*s < 0.20, *p*s > 0.66. Further, there was no significant change in self-reported fear between films C and D, *Wilk’s Λ* = 0.97, *F*(1, 92) = 2.53, *ηp^2^* = 0.03, *p* = 0.12. When comparing negative affect between film C and film D (and controlling for participant sex), there is no significant effect after correction, *Wilk’s Λ* = 0.94, *F*(1, 90) = 5.84, *p* = 0.02, *ηp^2^* = 0.06.

#### Physiological reactivity

Results comparing person-centered skin conductance reactivity (when controlling for participant sex) revealed no significant change in skin conductance between films A&B and films C&D or between film C and film D, *F*s < 0.22, *p*s > 0.64.

However, these analyses were also tested without participant sex included as a covariate, as sex was not significant related to any outcome variables within this analysis. Without covariates included, there is a significant change in EDA between films A&B and films C&D, *Wilk’s Λ* = 0.57, *F*(1,80) = 60.22, *p* < 0.001, *ηp^2^* = 0.43, such that EDA reactivity during films A&B (*M* = 0.02, *SD* = 0.04) was significantly lower than EDA reactivity indexed during films C&D after emotion regulation was introduced (*M* = 0.15, *SD* = 0.14), *MD* = −0.13, *p* < 0.001, 95% CI [−0.17, −0.10]. Further, when comparing films C and D there is a significant change in EDA from film C to film D, *Wilk’s Λ* = 0.66, *F*(1, 87) = 44.60, *p* < 0.001, *ηp^2^* = 0.34, such that EDA during film C (*M* = 0.12, *SD* = 0.13) was significantly lower than EDA during film D after specific regulatory strategies were introduced (*M* = 0.19, *SD* = 0.16), *MD* = −0.07, *p* < 0.001, 95% CI [−0.10, −0.05].

#### Facial expressions

Results of repeated measures ANCOVAs examining the relation between facial expressions between films A&B and films C&D (controlling for sex) did not reveal any significant differences for negative or fear facial expressions, *F*s < 0.16, *p*s > 0.69, and analyses comparing negative and fear facial expressions between films C and D also did not reveal significant change due to emotion regulation instruction of any kind, *F*s < 0.03, *p*s > 0.38.

#### Recovery periods

Although not pre-registered, we repeated these same analyses to explore differences in self-reported affect and facial expressions during *recovery* periods that immediately followed films A&B versus films C&D and did not find any significant differences, *F*s < 1.95, *p*s > 0.17. Further, there were no significant differences in self-reported affect, skin conductance, or facial expressions between recovery periods following film C and film D, *F*s < 2.71, *p*s > 0.10. However, there were significant differences in person-centered skin conductance [*Wilk’s Λ* = 0.52, *F*(1, 77) = 71.46, *ηp^2^* = 0.48, *p* < 0.001] between recovery after films A&B (*M* = −0.01, *SD* = 0.07) and films C&D (*M* = 0.15, *SD* = 0.14) such that recovery periods in C&D retained higher levels of skin conductance during recovery periods.

#### Emotion regulatory reports/strategies and emotion response output

*T*-tests and bivariate correlations explored self-reported and open-response emotion regulation strategies over time and in association to emotion responses (self-reported affect, skin conductance, facial expressions). Self-reported emotion regulation strategies during Film C were significantly related to self-reported emotion regulation strategies from the checklist during film D (*r* = 0.63, *p* < 0.001), suggesting that participants were significantly changing or increasing the number of strategies they were using after exposure to the checklist. The same was true for open-ended spoken emotion regulation strategies (*r* = 0.67, *p* < 0.001). However, there were no significant correlations between any self-reported checklist and any open-ended responses, *r*s < 0.20, *p*s > 0.06.

Number of open-response emotion regulation actions following film C (instruction only) was significantly negatively associated with skin conductance during film D, *r* = −0.27, *p* = 0.01. Number of open-response emotion regulation actions reported after film D (after checklist presentation) was related to skin conductance during film C, *r* = −0.21, *p* = 0.045, and film D, *r* = −0.25, *p* = 0.02. Further, number of emotion regulation actions reported during the open-response after film C was significantly positively related to negative facial expressions during the recovery period following film C, *r* = 0.25, *p* = 0.02, number of open-response strategies reported following film D was significantly positively related with negative facial expressions during film D, *r* = 0.24, *p* = 0.03, and the number of checklist items reported after film D was significantly positively related to both negative facial expressions during film D, *r = 0*.26, *p* = 0.02, the recovery period after film D, *r* = 0.23, *p* = 0.04. Importantly, neither self-reported nor open-response emotion regulation strategies were related to self-reported negative affect. In sum, greater open-response reports appeared to be associated with reduced autonomic arousal during films C and D as well as during recovery from each film, whereas greater checklist reports and some open-ended responses were positively associated with greater negative emotional facial expressions during and following each film.

*Post-hoc* exploratory analyses were conducted to investigate the concordance between self-reported and open-response emotion regulation strategies. Utilizing the four dominant regulatory strategy categories (i.e., reappraisal, distraction, acceptance, and rumination), paired samples *t*-tests revealed that rumination was the only strategy that did not evidence a significant difference across the different methods of data collection after Film C, *t*(93) = −0.96, *p* = 0.34, and Film D, *t*(92) = −0.90, *p* = 0.37. Next, utilization of an aggregate of open-ended responses and checklists of these primary strategies in Film C compared to Film D demonstrated a significant difference between Film C and Film D, where the average number of strategies reported was significantly greater after Film C (*M* = 2.48, SD = 1.17) compared to Film D (*M* = 2.16, SD = 1.16; *t*(92) = 2.56, *p* = 0.01).

### Moderation analyses

#### Trait negative affectivity

Results of analyses examining negative affectivity as a moderator in the relation between self-reported negative affect in films A&B and films C&D (while also controlling for participant sex) did not reveal a significant interaction, *Wilk’s Λ* = 0.99, *F*(1, 82) = 0.17, *ηp^2^* = 0.002, *p* = 0.68. However, when comparing negative affect between films C and D, there is a significant interaction, *Wilk’s Λ* = 0.87, *F*(1, 86) = 12.42, *ηp^2^* = 0.13, *p* < 0.001. This interaction was probed using two groups, one with negative affectivity greater than 1 SD above the mean and one with negative affectivity lower than 1 SD below the mean. This analysis suggests that only the group low in trait negative affectivity demonstrated significantly *lower* self-reported negative affect following film D compared to film C (see Figure 3).

**Figure 3:**
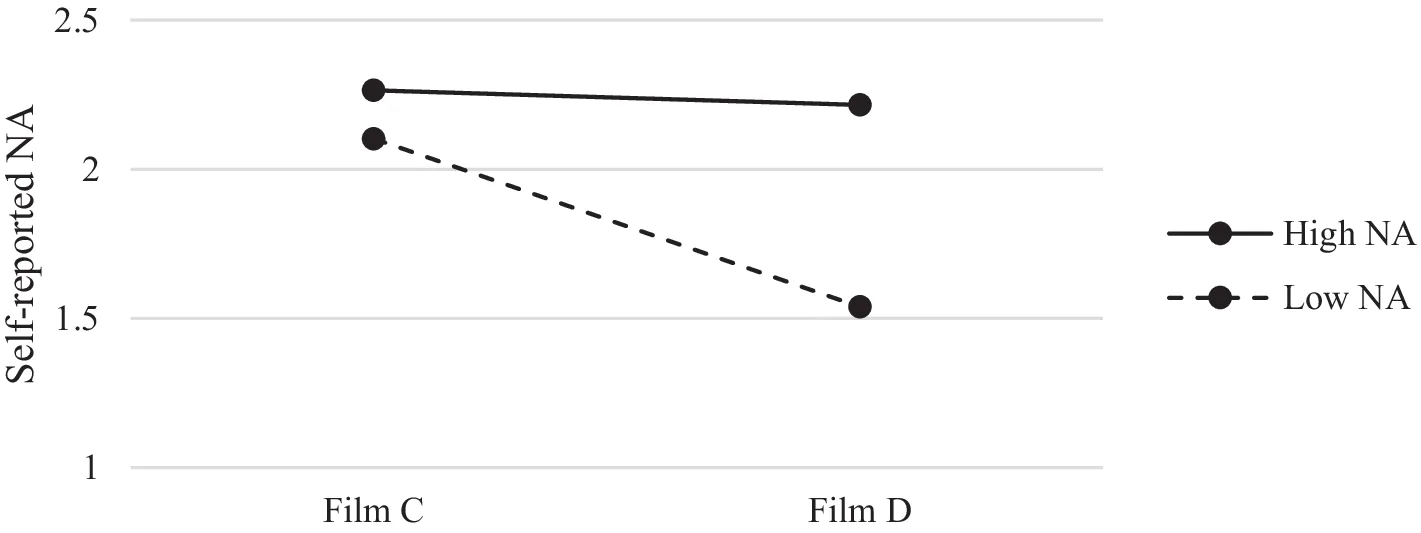
Interaction of time and trait negative affectivity on self-reported negative affect during films C and D.

There was no significant impact of negative affectivity on the change in self-reported fear between films A&B and films C&D or between films C and D, *F*s < 3.56, *p*s > 0.06, nor was there a significant impact of negative affectivity on the change in EDA from films A&B and films C&D or films C and D, *F*s < 1.54, *p*s > 0.22. Further, trait negative affectivity did not demonstrate a significant impact on change in negative facial expressions or fear expressions between films A&B and films C&D, *F*s < 0.65, *p*s > 0.43.

However, there is a significant effect of negative affectivity on negative facial expressions between films C and D, *Wilk’s Λ* = 0.95, *F*(1, 77) = 4.48, *ηp^2^* = 0.06, *p* = 0.04. This interaction was also probed using high and low negative affectivity groups, which suggested that the high negative affectivity group demonstrated *greater* negative facial expressions during film D compared to film C (see Figure 4). This finding was also significant for fear faces specifically, *Wilk’s Λ* = 0.94, *F*(1, 77) = 5.34, *ηp^2^* = 0.07, *p* = 0.02. This interaction demonstrated that the high trait negative affectivity group demonstrated greater fear facial expressions across films C and D than the low trait negative affectivity group (see Figure 5).

**Figure 4:**
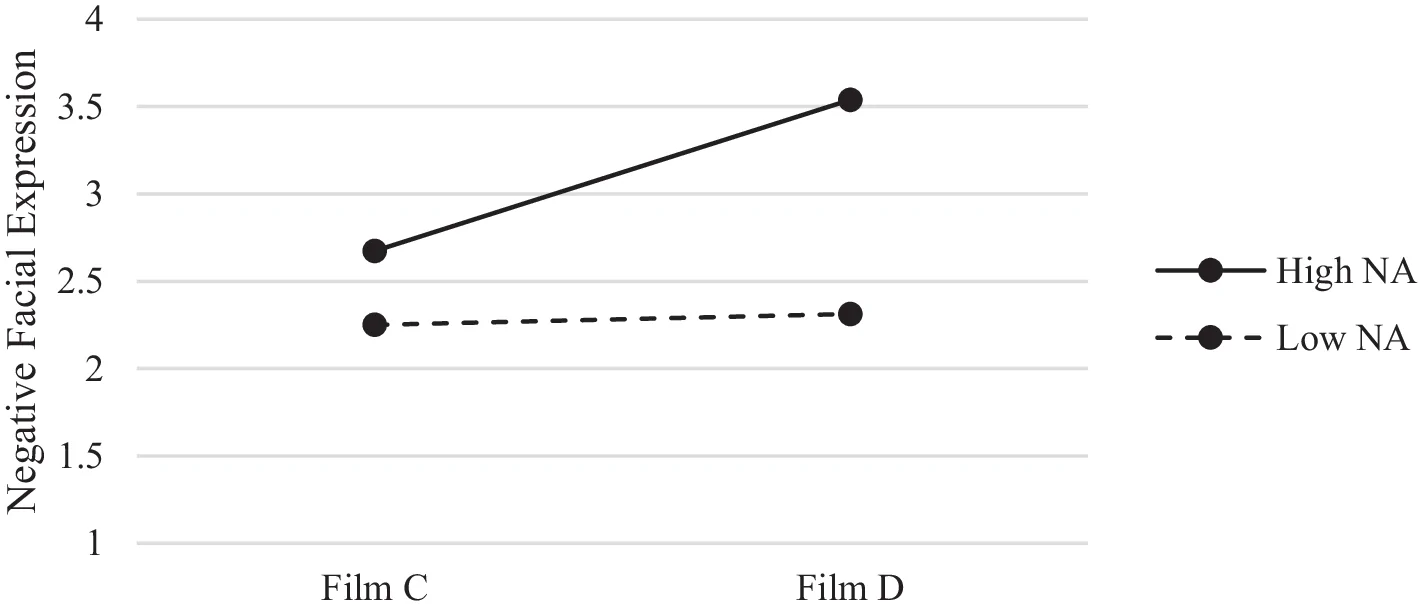
Interaction of time and trait negative affectivity on negative facial expresions during films C and D.

**Figure 5:**
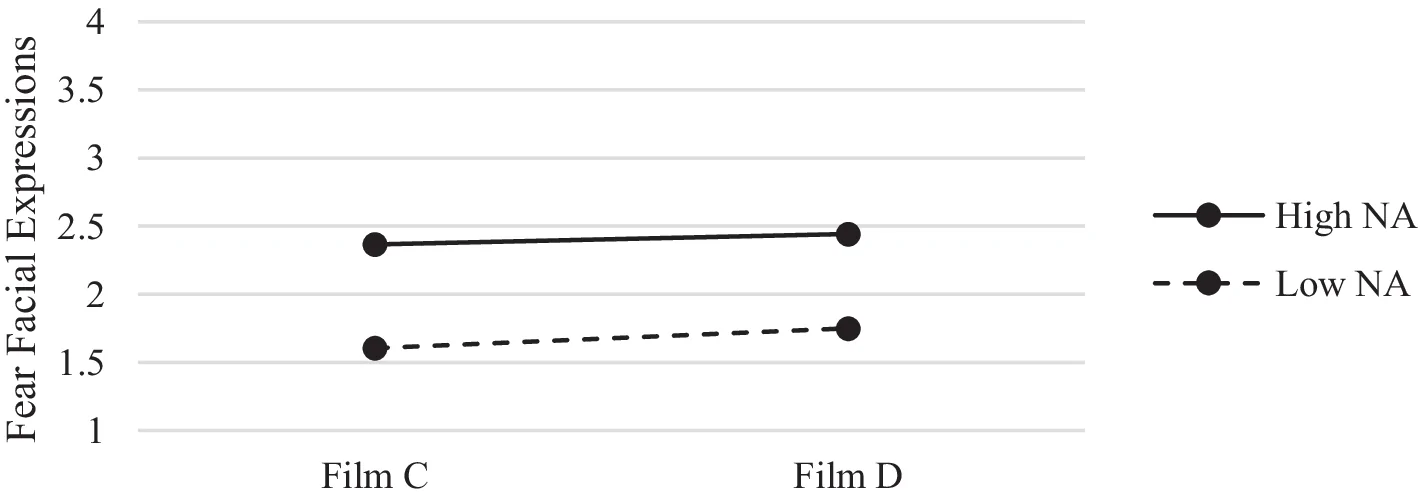
Interaction of time and trait negative affectivity on fear facial expressions during films C and D.

#### Response inhibition

Results of analyses examining interference on the negative block of the Stroop task as a moderator did not demonstrate any significant impact on emotion output (self-reported affect, *F*s < 0.81, *p*s > 0.37, skin conductance, *F*s < 0.16, *p*s > 0.69, or facial expressions, *F*s < 1.13, *p*s > 0.30). We tested these same effects using neutral interference scores and they were identical (see Supplemental Analyses).

## Discussion

Emotion regulation is a key process that facilitates daily navigation of life demands (Gross, 1998, 2015) and therefore it is not surprising that deficits in emotion regulation underly the development of the most common psychiatric disorders (Kring, 2008). Understanding the efficacy of prompting individuals with explicit demands to regulate their emotions is especially important, as this is a core feature of research but also of dominant psychotherapeutic interventions (e.g., Cognitive Behavioral Therapy, Beck, 1995; Emotion Regulation Therapy: Menin and Fresco, 2014; Dialectical Behavior Therapy, Linehan, 1993; Acceptance and Commitment Therapy, Hayes and Strosahl, 2005) used to treat emotion-related disorders such as depression, anxiety, and stress disorders. The present study sought to determine the impact of systematic increases in attention to emotion regulation on emotion responses while watching a series of fear-inducing films. Further, the present study also sought to investigate how key individual differences (i.e., trait negative affectivity, response inhibition) would impact efficacy of emotion regulation output under those circumstances.

The primary findings suggest that generally providing individuals with information about emotion regulation did not appear to have a substantial impact on self-reported experience, physiological response, or facial expression of emotion. Indeed, there was relatively little change in emotion output in response to films “A” and “B,” as compared to films “C” and “D” following the systematic introduction of emotion regulation, and the initiation of open-ended and strategy specific reports. This was despite significant associations between reports of strategy use and emotion responses assessed in-vivo during films “C” and “D.” However, additional analyses suggested that trait negative affectivity moderated the efficacy of responses to the emotion regulation information in meaningful ways. In particular, individuals high in trait negative affectivity were relatively unchanged in their self-reported negative emotion and (whereas individuals lower on this dimension reported significantly lower negative emotions) and evidenced *increases* in negative emotion facial expressions (including fear expressions specifically) after receiving specific strategy guidance (between films “C” and “D”). This finding has clear implications for interventions designed to train regulatory action in psychiatric patients, given that elevated trait negative affectivity represents a common dimension underlying most psychiatric disorders that are targeted in dominant psychotherapeutic treatments (Shackman et al., 2016; Barlow et al., 2017). Importantly, providing participants with a checklist of emotion regulation strategies also did not significantly impact physiological or facial emotional responses, nor did it appear to impact open-ended description of emotion regulation. However, participants did report lowered self-reported affect from film C (following emotion regulation information) to film D (following presentation of emotion regulation strategies) consistent with the explicit demand of the task. Finally, participant sex was significantly related to differences in self-reported affect, indicating that male participants were more responsive, demonstrating a significant decrease in reports of negative affect from film C to D, whereas this was not evident in female participants.

The results from this investigation are novel and do have potential implications for future research and for emotion regulation-related interventions. First, these findings appear broadly consistent with theory suggesting that emotion regulation is a combination of deliberate and implicit/automatic processes (Braunstein et al., 2017; Koole and Rothermund, 2011; Mauss et al., 2007) developed and honed throughout the lifespan. Indeed, emotion regulation can be measured as early as infancy (Bridges et al., 1997; Enlow et al., 2011; Feldman et al., 2004) and is continuously shaped through childhood and adolescence (Cole et al., 1994). However, by the time an individual reaches adulthood, it is likely their emotion regulatory repertoire has become largely automatic due to habitual emotion regulation strategy use over many years (Mauss et al., 2007). While the automation of this process may be adaptive in order to conserve cognitive resources, it makes measuring emotion regulation via self-report and intervening via psychoeducation more difficult. Indeed, our findings suggest that this may be particularly difficult for individuals with higher dispositional negative affect, and potentially the greatest need for support.

There are two key findings from this investigation that warrant much further explication. First, individuals high in trait negative affectivity demonstrated increases in negative facial expressions, in response to the fear-evoking films, *after* being presented with the emotion regulation checklist (Film D) compared to simply being provided with emotion regulation information (Film C) and that high trait negative affectivity was related to higher facial expressions across both films C and D compared to those with low trait negative affectivity. Further, low negative affectivity was related to a decrease in self-reported negative affect in film D compared to film C. These results are consistent with previous research suggesting that individuals with high trait negative affectivity may particularly struggle to regulate their emotions (Ng and Diener, 2009; Silverman et al., 2019; Yang et al., 2020) and this may be because the increased intensity of emotional responses (Norris et al., 2007) demands greater cognitive control to down-regulate (Dennis and Chen, 2007). For example, prior work has demonstrated that it is the interplay of the intensity of negative affect with cognitive control processes that can determine the reliance of maladaptive or adaptive regulatory responses in patients with common emotion-related disorders (Coifman and Aurora, 2022) and individuals with high trait negative affectivity (Coifman et al., 2018). Moreover, individuals with high trait negative affectivity may struggle to discriminate between their cognitive and physiological experience of emotions more than individuals low in trait negative affectivity (Riggio and Riggio, 2002). Indeed, prior evidence has suggested general deficits in the complexity of their negative emotional reports (Matt et al., 2016). Emotion regulation is complex and involves layers of cognitive, physiological, and automatic processes working together (Gross, 1998). Thus, shifting implicit demands to regulate to explicit demands to regulate (especially when this is inconsistent with how they habitually respond to emotion) may not be enough to impact the constellation of associated responses, including the felt experience of emotion, particularly for individuals high in trait negative affectivity. However, these findings should be considered with caution, particularly as these analyses were exploratory. Future research should further examine the impact of trait negative affectivity in the ability to downregulate negative emotions implicitly and explicitly.

These findings may be particularly relevant for individuals seeking psychotherapy, as successful implementation of newly learned strategies may depend on the individual’s ability to choose to engage in the new strategy in the moment and follow emotion regulation instructions during intense emotional experiences. This process requires engaging in self-regulation tasks such as (a) identifying the need for regulation, (b) deciding whether and how to regulate, and (c) enacting a strategy (Webb et al., 2012). This may be particularly difficult to do when faced with extreme emotions and their associated physiological reactions that may be counter to the emotion regulation strategy selected (e.g., choosing to not engage in avoidance during a time of high fear/anxiety). Many interventions focus on explicit emotion regulation goals and processes and emphasize skill use outside of session and practice in order to better learn to implement the emotion regulation skills. This research suggests repeated practice and skill building may be necessary for effective use of these skills. However, the degree to which this has been empirically investigated remains quite limited. Interestingly, exploratory analyses examining the interaction of individual differences on emotion regulation efficacy demonstrated no significant differences in emotional response based on cognitive resources related to response inhibition. Previous research on the impact of response inhibition on emotion regulation is mixed, with recent work illustrating that working memory may play a more important role in emotion regulation, (Coifman and Aurora, 2022; Coifman et al., 2019). However, as the present study focused on responses to fear, it is important to note that implicit emotion regulation goals and processes do also play a role in common therapies for anxiety and trauma disorders (i.e., exposure therapy; Craske et al., 2012; Foa and McLean, 2016). Exposure therapy relies on emotional extinction, which is considered an implicit emotion regulation process. Because therapies utilize a range of emotion regulation strategies, it is important to understand who is able to benefit from these processes and what role awareness and goal-driven regulation play in the effectiveness of these treatments.

There are limitations to this work to be considered. First, the sample used in the present study was relatively small and non-diverse undergraduate sample. The sample was also not evaluated for or excluded on the basis of psychiatric disorders. Given the implications of the present study, future research should aim to investigate individual differences in the efficacy of emotion regulation within a more diverse sample, including variable ages, psychiatric presentations, and/or developmental stage. Replicating and further explicating these findings in larger and in clinical samples may also provide important information about the individual differences in responding to explicit and implicit emotion regulation demands and further insight into the processes affecting, effective emotion regulation. An additional limitation was the precise approach of introducing emotion regulation. Here, we aimed to orient participants first to the topic and their own actions, and next to various ways of regulating. This is decidedly distinct from actual instruction in emotion regulation. However, given that commonly patients are instructed in regulation in therapy sessions and then expected to translate this into real-life use, we felt that the approach suitable. That being said, future replications of this research should consider a variety of methods to introduce emotion regulatory action, to test their impacts. Future research may also benefit from further examination of the reported emotion regulation strategies, as this may provide more insight into the use of these strategies implicitly and explicitly. Another important limitation of this study design was the use of fear films explicitly. Results of this study may not generalize to other negative emotional experiences (i.e., disgust, sadness, anger). Further, due to the nature of this experimental manipulation, it is important to note that time and repeated exposure to fear-inducing films may be an alternative explanation for the findings in this study. It is possible that the exposure to the fourth fear film may have had an additional negative effect on participants compared to the second or third film, making it difficult to conclude that results were due to the emotion regulation priming and not to increasing negative affect over time. Indeed, this effect might even be more significant for individuals higher in trait negative affectivity. Future research may consider utilizing more externally valid methodologies (i.e., ecological momentary assessment) to better understand how these processes unfold in daily life. In-vivo data collection may allow us to further examine the emotion regulatory process and gather critical contextual information (i.e., social context, changing emotion regulation goals; Colombo et al., 2020; Swerdlow et al., 2022).

Importantly, however, this research also had a number of key strengths. In particular, the use of a carefully conceived within-person assessment paradigm that was able to index emotion response output broadly (across subjective and objective indicators) and that allowed for thorough assessment of baseline spontaneous emotional responses (prior to emotion regulation introduction). Moreover, that we considered emotion regulation as first attention to processes and then report of specific actions (using both open-ended and checklist reporting) allowed us to systematically examine how each step impacts emotion response output. Indeed, that we found that emotion response output was largely unchanged between films A/B and films C/D suggests that simply introducing the idea of emotion regulation and drawing the individual’s attention to it is insufficient to change emotion responses. That being said, participants’ report of regulatory actions (both open-ended and strategy selection) was associated with emotion output in films C and D, indicating that greater regulatory strategy use was resulting in reduced emotional responding to the fear films. Moreover, the present study highlights the importance of evaluating methods of experimentally measuring and manipulating emotion regulation. Because emotion regulation research relies so heavily on explicit emotion regulation and self-reported emotion regulation, it is critical to determine how successfully participants are able to report on their emotional experience and how effective they are able to be at regulating their emotions with explicit goals to do so.

In sum, in this investigation, we systematically evaluated the impact of attention to emotion regulatory actions and then strategies used during a series of emotionally evocative films in college students. The key results suggest that overall, introducing attention to emotion regulation did not alter emotion response output. However, the results provide preliminary support for the impact of individual differences in trait negative affectivity on emotion regulation efficacy. Indeed, for those with elevated trait negative affective, there was little evidence of responses to emotion regulatory strategy suggestion and some evidence of increases in negative (and fear) emotion. Whereas for those individuals low in negative affectivity, changes in emotion responses were more clear. Given the likelihood that individuals seeking psychotherapy are high in trait negative affectivity, future research examining the how to facilitate greater responsivity to changing demands to regulate emotions and/or the implantation of new, more effective, strategies is needed to continue to inform psychiatric interventions and research on emotion regulation broadly construed.

## Data Availability

The datasets presented in this study can be found in online repositories. The names of the repository/repositories and accession number(s) can be found below: these analyses were pre-registered (https://osf.io/wzcy8).

## Footnotes

1
https://osf.io/wzcy8

2 https://osf.io/wzcy8.
