# Supplementary material for: Who regulates when told? A systematic investigation of implicit to explicit emotion regulation
Source: Front Psychol. 2026 Sep 15;17:1852337. doi: 10.3389/fpsyg.2026.1852337 (PMC13619395; doi:10.3389/fpsyg.2026.1852337)
Supplement: Supplementary file 1 [file Table_1.DOCX]

**Supplemental Materials**

**Table of Contents**

***Pilot Study (Table S1)	2***

***Study Materials	4***

***Table S2	14***

***Table S3	15***

***Table S4	15***

***Table S5	15***

***Table S6	16***

***Neutral Stroop Results	16***

**Pilot Study**

In order to develop the fear reactivity task for the current investigation, a pilot study was first conducted to assess the efficacy of the video stimuli. For the pilot study, four fear eliciting video clips were selected from publicly available clips. In the four selected videos, the following content was depicted: 1) a doctor is examining an infected wound on the back of a young woman. At the end of the clip, spiders crawl out of the wound and the young woman screams (Fear Clinic, Anchor Bay Entertainment, 2014), 2) a woman wakes up to find a demon- like character next to her in bed (Bedfellows, www.youtube.com), 3) in a dark house, arms and hands extend through the walls and ceiling forming a monster or demon-like creature (Real Demons, www.youtube.com), 4) a large spider suddenly jumps down from the ceiling on a man standing on a ladder (Spider Attacks Daddy, [www.youtube.com](http://www.youtube.com)).

All selected video clips were evaluated as part of an online pilot study, consistent with well-established methodology for choosing video stimuli (Gross & Levenson, 1995). A sample of n=318 college students was recruited from the Psychology department subject pool for the pilot study. Data was dropped from a total of n=47 participants due to non-completion of the study (n=3) and poor engagement with the video content (n=44) (detailed below) thus, the final pilot sample consisted of n=271 participants (78.6% female, 85.2% Caucasian, age: M=19.58, SD=1.73 years). After giving informed consent, participants were asked to provide demographic information, and then they viewed the above detailed video clips (presented in random order). Following each video clip, participants were asked to report on their emotional experience by rating emotion-words on a 7-point Likert scale. Emotion-words were of both negative (fear, sadness, disgust, guilt, distress, anger) and positive (happiness, enjoyment, amusement, affection, relief) valence. These emotion-words demonstrated sufficient internal consistency in this sample (negative emotion experience in response to fear context a=.90, and positive emotion experience in response to positive emotion context; a=.92). Following each video clip, participants were also asked to answer one question regarding the content of the video clip, in order to ensure engagement with the video. For example, following the “Bedfellows” video clip, participants were asked “In what room did most of the action in this clip take place?” (correct answer: “the bedroom”). Participants who did not answer the questions correctly were dropped from the study (n=44). Participants received course credit upon completion of the study.

**Results**

In order to test the efficacy of the selected video clips in eliciting the target emotion (fear), responses were analyzed following the example of Gross and Levenson (1995) (c.f. Gilman et al., 2017). A “success index” was established for each video clip by utilizing the standardized mean *intensity* of the clip for the target emotion (mean rating of *fear* for each fear video) and a *discreteness* value (percentage of participants who rated the target emotion, *fear*, at least one point above all non-target emotions). Mean intensity scores and mean discreteness scores were then standardized as z-scores and the sum of these two z-scores represents the success index of each video clip, relative to other video clips assessed for the same target emotion (see Table 1). Video clips with the highest success rate have been suggested for use (Gross & Levenson, 1995).

Table S1. Pilot study success indices for each video

**Study Materials**

**Affect Rating Sheet:**

**What are you feeling now?**

None	Mild	Moderate	Strong

| 1. Interest ……………………….. | 1 | 2 | 3 | 4 | 5 | 6 | 7 |
| --- | --- | --- | --- | --- | --- | --- | --- |
| 2. Fear…………………………… | 1 | 2 | 3 | 4 | 5 | 6 | 7 |
| 3. Relief…………………………. | 1 | 2 | 3 | 4 | 5 | 6 | 7 |
| 4. Sadness……………………….. | 1 | 2 | 3 | 4 | 5 | 6 | 7 |
| 5. Enjoyment……………………. | 1 | 2 | 3 | 4 | 5 | 6 | 7 |
| 6. Distress………………………. | 1 | 2 | 3 | 4 | 5 | 6 | 7 |
| 7. Surprise………………………. | 1 | 2 | 3 | 4 | 5 | 6 | 7 |
| 8. Guilt…………………………... | 1 | 2 | 3 | 4 | 5 | 6 | 7 |
| 9. Happiness…………………….. | 1 | 2 | 3 | 4 | 5 | 6 | 7 |
| 10. Anger………………………….. | 1 | 2 | 3 | 4 | 5 | 6 | 7 |
| 11. Amusement…………………… | 1 | 2 | 3 | 4 | 5 | 6 | 7 |
| 12. Disgust……………………….. | 1 | 2 | 3 | 4 | 5 | 6 | 7 |
| 13. Affection….…………………... | 1 | 2 | 3 | 4 | 5 | 6 | 7 |
| 14. Boredom……………………… | 1 | 2 | 3 | 4 | 5 | 6 | 7 |
| 15. Fatigue………………………… | 1 | 2 | 3 | 4 | 5 | 6 | 7 |

**Emotion Regulation Checklist:**

**Please check off any of the below statements that is a good representation of what you did in response to the video.**

______	I accepted my emotions

______	I found an activity to keep myself busy and distracted

______	I thought over and over again about the situation and my feelings

______	I made a plan to make the situation better

______	I reminded myself that things could be worse

______	I thought about how I could become stronger and learn from this situation

______	I thought about all the different things in my life that this situation would impact

______	I thought about how this situation was my fault

______	I thought about all the other things that have happened to me in addition to this

______	I thought about how the situation was someone else’s fault

______	I controlled my emotions by not showing them

______	I thought of something pleasant instead of what had happened

______	I thought about the situation in a different way

______	I went to sleep

______	I ignored my feelings

______	I just acted like the situation had never happened at all

**Symptom Checklist 90 – R, SCL-90-R (Derogatis, 1983)**

Below is a list of problems people sometimes have. Please read each one carefully and circle the number to the right that best describes HOW MUCH THAT PROBLEM HAS DISTRESSED OR BOTHERED YOU DURING THE PAST 7 DAYS INCLUDING TODAY. Circle only one number for each problem and do not skip any items. If you change your mind, erase your first mark carefully.

| How much were you distressed by: | Not At All | A Little | Moderate | Quite A Bit | Extreme |
| --- | --- | --- | --- | --- | --- |
| 1. Nervousness or shakiness inside. | 0 | 1 | 2 | 3 | 4 |
| 2. Loss of sexual interest or pleasure. | 0 | 1 | 2 | 3 | 4 |
| 3. Feeling easily annoyed or irritated. | 0 | 1 | 2 | 3 | 4 |
| 4. Feeling low in energy or slowed down. | 0 | 1 | 2 | 3 | 4 |
| 5. Thoughts of ending your life. | 0 | 1 | 2 | 3 | 4 |
| 6. Trembling. | 0 | 1 | 2 | 3 | 4 |
| 7. Crying easily. | 0 | 1 | 2 | 3 | 4 |
| 8. Feeling of being caught or trapped. | 0 | 1 | 2 | 3 | 4 |
| 9. Suddenly scared for no reason. | 0 | 1 | 2 | 3 | 4 |
| 10. Temper outbursts that you could not control. | 0 | 1 | 2 | 3 | 4 |
| 11. Blaming yourself for things. | 0 | 1 | 2 | 3 | 4 |
| 12. Feeling lonely. | 0 | 1 | 2 | 3 | 4 |
| 13. Feeling blue. | 0 | 1 | 2 | 3 | 4 |
| 14. Worrying too much about things. | 0 | 1 | 2 | 3 | 4 |
| 15. Feeling no interest in things. | 0 | 1 | 2 | 3 | 4 |
| 16. Feeling fearful. | 0 | 1 | 2 | 3 | 4 |
| 17. Heart pounding or racing. | 0 | 1 | 2 | 3 | 4 |
| 18. Feeling hopeless about the future. | 0 | 1 | 2 | 3 | 4 |
| 19. Feeling tense or keyed up. | 0 | 1 | 2 | 3 | 4 |
| 20. Having urges to beat, injure, or harm someone. | 0 | 1 | 2 | 3 | 4 |
| 21. Having urges to break or smash things. | 0 | 1 | 2 | 3 | 4 |
| 22. Feeling everything is an effort. | 0 | 1 | 2 | 3 | 4 |
| 23. Spells of terror or panic. | 0 | 1 | 2 | 3 | 4 |
| 24. Getting into frequent arguments. | 0 | 1 | 2 | 3 | 4 |
| 25. Feeling so restless you couldn’t sit still. | 0 | 1 | 2 | 3 | 4 |
| 26. Feelings of worthlessness. | 0 | 1 | 2 | 3 | 4 |
| 27. The feeling that something bad is going to happen to you. | 0 | 1 | 2 | 3 | 4 |
| 28. Shooting or throwing things. | 0 | 1 | 2 | 3 | 4 |
| 29. Thoughts and images of a frightening nature. | 0 | 1 | 2 | 3 | 4 |

**State-Trait Anxiety Inventory, STAI (Spielberger et al, 1970)**

Instructions: A number of statements which people have used to describe themselves are given below. Please read each statement and then circle the appropriate value to the right of the statement to indicate how you *generally* feel. There are no right or wrong answers. Do not spend too much time on any one statement but give the answer that seems to describe how you *generally* feel.

|  | Almost Never | Sometimes | Often | Almost Always |
| --- | --- | --- | --- | --- |
| 1. I feel pleasant | 1 | 2 | 3 | 4 |
| 2. I feel nervous and restless | 1 | 2 | 3 | 4 |
| 3. I feel satisfied with myself | 1 | 2 | 3 | 4 |
| 4. I wish I could be as happy as others seem to be | 1 | 2 | 3 | 4 |
| 5. I feel like a failure | 1 | 2 | 3 | 4 |
| 6. I feel rested | 1 | 2 | 3 | 4 |
| 7. I am “calm, cool, and collected” | 1 | 2 | 3 | 4 |
| 8. I feel that difficulties are piling up so that I cannot overcome them | 1 | 2 | 3 | 4 |
| 9. I worry too much over something that really doesn’t matter | 1 | 2 | 3 | 4 |
| 10. I am happy | 1 | 2 | 3 | 4 |
| 11. I have disturbing thoughts | 1 | 2 | 3 | 4 |
| 12. I lack self-confidence | 1 | 2 | 3 | 4 |
| 13. I feel secure | 1 | 2 | 3 | 4 |
| 14. I make decisions easily | 1 | 2 | 3 | 4 |
| 15. I feel inadequate | 1 | 2 | 3 | 4 |
| 16. I am content | 1 | 2 | 3 | 4 |
| 17. Some unimportant thought runs through my mind and bothers me | 1 | 2 | 3 | 4 |
| 18. I take disappointments so keenly that I can’t put them out of my mind | 1 | 2 | 3 | 4 |
| 19. I am a steady person | 1 | 2 | 3 | 4 |
| 20. I get in a state of tension or turmoil as I think over my recent concerns and interests | 1 | 2 | 3 | 4 |

**BIS/BAS scales (Carver, C. S., & White, T. L., 1994)**

Each item of this questionnaire is a statement that a person may either agree with or disagree with.  For each item, indicate how much you agree or disagree with what the item says.  Please respond to all the items; do not leave any blank.  Choose only one response to each statement.  Please be as accurate and honest as you can be.  Respond to each item as if it were the only item.  That is, don't worry about being "consistent" in your responses.  Choose from the following four response options:

  1 = very true for me 
  2 = somewhat true for me 
  3 = somewhat false for me 
  4 = very false for me

|  | Very true (1) | Somewhat True (2) | Somewhat False (3) | Very False (4) |
| --- | --- | --- | --- | --- |
| 1. A person’s family is the most important thing in my life. | 1 | 2 | 3 | 4 |
| 2. Even if something bad is about to happen to me, I rarely experience fear or nervousness. | 1 | 2 | 3 | 4 |
| 3. I go out of my way to get things I want. | 1 | 2 | 3 | 4 |
| 4. When I’m doing well at something I love to keep at it. | 1 | 2 | 3 | 4 |
| 5. I’m always willing to try something new if I think it will be fun. | 1 | 2 | 3 | 4 |
| 6. How I dress is important to me. | 1 | 2 | 3 | 4 |
| 7. When I get something I want, I feel excited and energized. | 1 | 2 | 3 | 4 |
| 8. Criticism or scolding hurts me quite a bit. | 1 | 2 | 3 | 4 |
| 9. When I want something I usually go all-out to get it. | 1 | 2 | 3 | 4 |
| 10. I will often do things for no other reason than that they might be fun. | 1 | 2 | 3 | 4 |
| 11. It’s hard for me to find the time to do things such as get a haircut | 1 | 2 | 3 | 4 |
| 12. If I see a chance to get something I want I move on it right away. | 1 | 2 | 3 | 4 |
| 13. I feel pretty worried or upset when I think or know somebody is angry at me. | 1 | 2 | 3 | 4 |
| 14. When I see an opportunity for something I like I get excited right away. | 1 | 2 | 3 | 4 |
| 15. I often act on the spur of the moment. | 1 | 2 | 3 | 4 |
| 16. If I think something unpleasant is going to happen I usually get pretty “worked up”. | 1 | 2 | 3 | 4 |
| 17. I often wonder why people act the way they do | 1 | 2 | 3 | 4 |
| 18. When good things happen to me, it affects me strongly. | 1 | 2 | 3 | 4 |
| 19. I feel worried when I think I have done poorly at something important. | 1 | 2 | 3 | 4 |
| 20. I crave excitement and new sensations. | 1 | 2 | 3 | 4 |
| 21. When I go after something I use a “no holds barred” approach. | 1 | 2 | 3 | 4 |
| 22. I have very few fears compared to my friends. | 1 | 2 | 3 | 4 |
| 23. It would excite me to win a contest. | 1 | 2 | 3 | 4 |
| 24. I worry about making mistakes. | 1 | 2 | 3 | 4 |

| Table S2. Missing data | | |
| --- | --- | --- |
| Total number of participants with missing data | Type of missing data | Reasons for missing data |
| 4 | All study data | 1. Participant did not speak English fluently (n=1) 2. Participant ended session early (n=2) 3. Participant previous participated in another study with the same stimuli (n=1) |
| 18 | EDA signal | 1. Equipment malfunction and/or administrator error (n=13) 2. Participant was a non-responder with low natural skin conductance (n=5) |
| 3 | Self-reported affect | 1. Participant misunderstood instructions when filling out affect ratings (n=3) |
| 4 | Emotion expression | 1. Video recording error (n=3) 2. Participant asked to not be recorded (n=1) |
| 2 | Stroop | 1. Administrator error (n=1) 2. Participant was color blind (n=1) |

| Table S3. Means and standard deviations of self-reported negative affect across all segments. | |
| --- | --- |
| **Segment** | **M (SD)** |
| Baseline | 1.07 (.25) |
| Film A | 2.00 (.89) |
| Film A Recovery | 1.52 (.80) |
| Film B | 1.91 (.87) |
| Film B Recovery | 1.46 (.62) |
| Film C | 2.16 (.83) |
| Film C Recovery | 1.48 (.57) |
| Film D | 2.08 (.90) |
| Film D Recovery | 1.50 (.61) |

| Table S4. Means and standard deviations of SCR across all segments. | |
| --- | --- |
| **Segment** | **M (SD)** |
| Baseline | 3.38 (2.86) |
| Film A | 4.31 (3.16) |
| Film A Recovery | 4.14 (3.12) |
| Film B | 4.84 (3.45) |
| Film B Recovery | 4.93 (3.33) |
| Film C | 5.23 (3.61) |
| Film C Recovery | 5.53 (3.53) |
| Film D | 5.91 (3.84) |
| Film D Recovery | 5.78 (3.69) |
| Note. Values presented are raw scores prior to transformation or person-centering. | |

| Table S5. Means and standard deviations of negative facial expressions across all segments. | |
| --- | --- |
| **Segment** | **M (SD)** |
| Baseline | 1.49 (.58) |
| Film A | 2.82 (1.47) |
| Film A Recovery | 1.84 (.76) |
| Film B | 3.17 (1.53) |
| Film B Recovery | 1.93 (.78) |
| Film C | 3.23 (1.59) |
| Film C Recovery | 1.83 (.65) |
| Film D | 3.11 (1.48) |
| Film D Recovery | 1.79 (.58) |

| Table S6. Stroop task scores | | | |
| --- | --- | --- | --- |
|  | **Congruent** | **Incongruent** | **Interference score** |
| **Task** | **M (SD)** | **M (SD)** | **M (SD)** |
| **Color-word Stroop** | 748.48 (99.85) | 791.43 (109.87) | 43.39 (49.04) |
| **Emotional Color-word Stroop** |  |  |  |
| Neutral condition | 752.73 (127.89) | 812.99 (137.53) | 60.26 (79.19) |
| Negative condition | 927.28 (223.47) | 983.42 (241.20) | 56.13 (127.36) |
| Positive condition | 768.92 (130.58) | 834.02 (151.25) | 64.92 (90.35) |

**Neutral Block Response Inhibition Findings**

Results of analyses examining interference on the neutral block of the stroop task as a moderator did not demonstrate any significant impact on self-reported affect, *F*s < .86, *p*s > .35, skin conductance, *F*s < 1.24, *p*s > .09, or facial expressions, *F*s < 1.36, *p*s > .43.

**Fear Results**

**Self-reported fear**

Results of the repeated measures ANCOVA examining the change in self-reported between films A&B and C&D were not significant, *F*s < .20, *p*s > .66. Further, there was no significant change in self-reported fear between films C and D, *Wilk's Λ* = .97, *F*(1, 92) = 2.53, *ηp^2^* = .03, *p* = .12

**Fear facial expressions**

Results of repeated measures ANCOVAs examining the relation between fear facial expressions between films A&B and films C&D (controlling for sex) did not reveal any significant differences, *F*s < .16, *p*s > .69, and analyses comparing fear facial expressions between films C and D also did not reveal significant change due to emotion regulation instruction of any kind, *F*s < .03, *p*s > .38.

**Trait negative affectivity**

There was no significant impact of negative affectivity on the change in self-reported fear between films A&B and films C&D or between films C and D, *F*s < 3.56, *p*s > .06. Further, trait negative affectivity did not demonstrate a significant impact on change in fear expressions between films A&B and films C&D, *F*s < .65, *p*s > .43. However, there is a significant effect of negative affectivity on fear facial expressions between films C and D, *Wilk’s Λ* = .94, *F*(1, 77) = 5.34, *ηp^2^* = .07, *p* = .02. This interaction demonstrated that the high trait negative affectivity group demonstrated greater fear facial expressions between films C and D than the low trait negative affectivity group (see Figure S6).
